# Presence-absence of plant habitat specialists in 15 patches of dry calcareous grassland

**DOI:** 10.3897/BDJ.10.e94057

**Published:** 2022-11-17

**Authors:** Eva Lieungh, Kristina Bjureke, Marianne Evju, Rukaya Sarah Johaadien, Siri Lie Olsen, Olav Skarpaas, Odd Egil Stabbetorp, Anders Kvalvåg Wollan

**Affiliations:** 1 Natural History Museum, University of Oslo, Oslo, Norway Natural History Museum, University of Oslo Oslo Norway; 2 Norwegian Institute for Nature Research, Oslo, Norway Norwegian Institute for Nature Research Oslo Norway; 3 Norwegian University of Life Sciences, Ås, Norway Norwegian University of Life Sciences Ås Norway

**Keywords:** sampling-event, vascular plants, specialist species, presence-absence data, calcareous grassland, habitat patch, GBIF

## Abstract

**Background:**

Dry grasslands on calcareous bedrock in warm climates around the Oslo Fjord are naturally fragmented biodiversity hotspots. This habitat geographically coincides with the most densely populated area of Norway. Many habitat specialists, along with the habitat itself, are red-listed because of land-use change, forest encroachment, and invasive species that cause habitat loss and greater isolation of remaining patches. To ensure effective conservation, data on species presences and absences are necessary to quantify states, changes, and extinction risks in specific populations and habitat patches.

**New information:**

We present presence-absence data of 49 vascular plant species in 15 patches of dry calcareous grassland habitat, surveyed in 2009, 2019, and in 2020. The species are considered to be habitat specialists and, thus, unlikely to occur between the patches.

## Introduction

Norway’s most densely populated area coincides geographically with a national biodiversity hotspot. Warm climates and calcareous bedrock have created dry grasslands in places with shallow soil and historical land-use practices. These grasslands are naturally fragmented, but are now Red-listed along with several habitat specialists that depend on them. Land-use change, forest encroachment, and invasive species cause habitat loss and greater isolation of remaining patches (Evju et al. 2018).

We present presence-absence data for 49 vascular plant species in 15 mapped patches of dry calcareous grassland habitat surveyed in three growing seasons. The data combine surveys from 2009, 2019, and 2020. The 2019 and 2020 surveys are repeated surveys of a subset of data described in previous publications (Wollan et al. 2011, Bakkestuen et al. 2014, Evju et al. 2015, Evju and Sverdrup-Thygeson 2016). As the species are considered to be habitat specialists, they are unlikely to occur between the patches (Evju et al. 2015). Although substantial habitat loss has occurred in the past ~ 80 years (Evju et al. 2016), dry calcareous grasslands occur sparsely and fragmented by nature. Therefore, it is plausible that the populations of habitat specialists, until recently, were in approximate equilibrium with their environment.

Dry calcareous grasslands are characterised by drought-tolerant grasses, forbs, shrubs and occasionally trees. Though originally defined as lacking tree cover, the borders are transient between naturally treeless and potentially forested semi-natural habitats kept open by grazing. The surveyed habitat patches also include semi-natural vegetation where dry pine forest has potential to grow. Interannual variability plays an important role, as occasional intense droughts keep competitive vegetation away from the driest patches. For example, the summer of 2018 was exceptionally dry and hot, and visibly impacted the vegetation the following years. Many drought-intolerant plants and young trees died, and some of the habitat specialists came back in full vigour the next season.

Some patches of dry calcareous grasslands, like those presented here, are protected inside nature reserves. A combination of appropriate theory and good data can provide insights into extinction risk scenarios and guide choices between alternative conservation strategies. Ecological theory, such as island biogeography and metapopulation and metacommunity theory, can provide hypotheses and general predictions. Good data, including absence records, are necessary to test these hypotheses and provide insight into specific populations and habitat patches. The present dataset was collected to study hypotheses of turnover and persistence in the metapopulations of habitat specialists and to quantify states and changes in a habitat of considerable conservation interest. As all the patches are on the same island, they form a closed system where species may persist, become extinct, or be colonised from neighbouring patches or patches on different islands. The dataset builds on a study design set up to monitor this hotspot habitat (Bakkestuen et al. 2014), following their habitat patch delineation and definitions of habitat specialist species.

## General description

### Purpose

The data from 2009 are a subset of the ARKO project data (Wollan et al. 2011) and the data from 2019 and 2020 were collected as part of EL's PhD project.

## Project description

### Title

Dynamic distribution modelling

### Personnel

Eva Lieungh

### Funding

Natural History Museum, University of Oslo, the Norwegian Ministry of Education and Research

## Sampling methods

### Study extent

Field surveys were carried out in 2009, 2019, and 2020. All vascular plant species inside the 15 habitat patches were recorded in 2009 and 2019, but only the habitat specialists' occurrences are reported in this dataset. In 2020, occurrence was only recorded for the 49 habitat specialists.

### Sampling description

Fieldwork in 2009 was carried out by KB, OES, and AKW, all experienced botanists. Potential locations of dry calcareous grassland had been identified by aerial photos and distribution modelling. The patch locations were then visited by the fieldwork team and either discarded or confirmed and delimited. Polygon coordinate points were recorded using a handheld GPS with an estimated accuracy of 1-10 m. All three in the team looked for species in the same patch, one being in charge of writing down occurrences. When no new species were found, all three tried to find one more species each before declaring the list complete. Species lists per patch were noted in a notebook. Fieldwork in the 2019 and 2020 surveys was carried out by EL alone. Patches were found using a map with semi-transparent polygons over a recent aerial photo. Species presences were recorded in a spreadsheet on a smartphone. Patches were inventoried by carefully walking back and forth across the polygon to cover the entire area. EL took pictures of each patch. EL also took pictures of uncertain species with the iNaturalist (iNaturalist 2008) app to aid identification after fieldwork as it is illegal to collect plants inside the protected area.

### Quality control

We took several steps to quality-check the data. Each occurrence was evaluated manually to ensure the highest possible quality of data: after data collection was complete in 2019 and 2020, each observation was checked against the data from the previous year(s), occurrences in GBIF, and pictures taken during fieldwork. Some uncertainty was resolved using pictures stored in iNaturalist, where pictures were stored for individual plants along with their coordinates and other metadata. Some of these pictures were sent to OS, SLO and other experts to confirm uncertain species identifications. Additional trips to Gressholmen by EL along with SLO were carried out in September 2019, and alone in June 2020, to check uncertain presences and suspected false absences.

### Step description

For 2009 and 2019, there are more data available than described here. These data include all vascular plant species, not just the specialists, and some additional descriptive variables for each patch.

In spite of quality control, some uncertainty remains. Delineating polygons inevitably involves uncertainties, and GPS polygon boundaries were not physically delimited in the field. In addition, changes in patch size may have occurred between 2009 and 2019, due to regrowth or other factors. To a lesser degree, this is also an issue between the 2019 and 2020 surveys. False absences may occur in the dataset, which is common in field surveys (Morrison 2016), either from incomplete survey effort or because the species was not detectable at the time of fieldwork. As flowering phenology varies, early-flowering species (e.g. *Veronicaarvensis*, *Drabaverna*) are especially susceptible to being omitted, even though special care was taken to look for them. Misidentification could also occur, though the availability of previous data and use of trained botanists with expert knowledge of the local flora should minimise this risk.

## Geographic coverage

### Description

Calcareous grasslands occur naturally fragmented on shallow soils by the Oslo Fjord, mainly on exposed and/or grazed Cambrian–Silurian marine sedimentary rock. The climate is relatively warm, with an annual temperature of 5.7°C (Aune 1993) and annual precipitation of 763 mm (Førland 1993). The area is amongst the most species-rich in Norway, as the combination of calcareous bedrock and warm summers is rare and hosts species that are mostly found further south in Europe.

**Figure 1.**
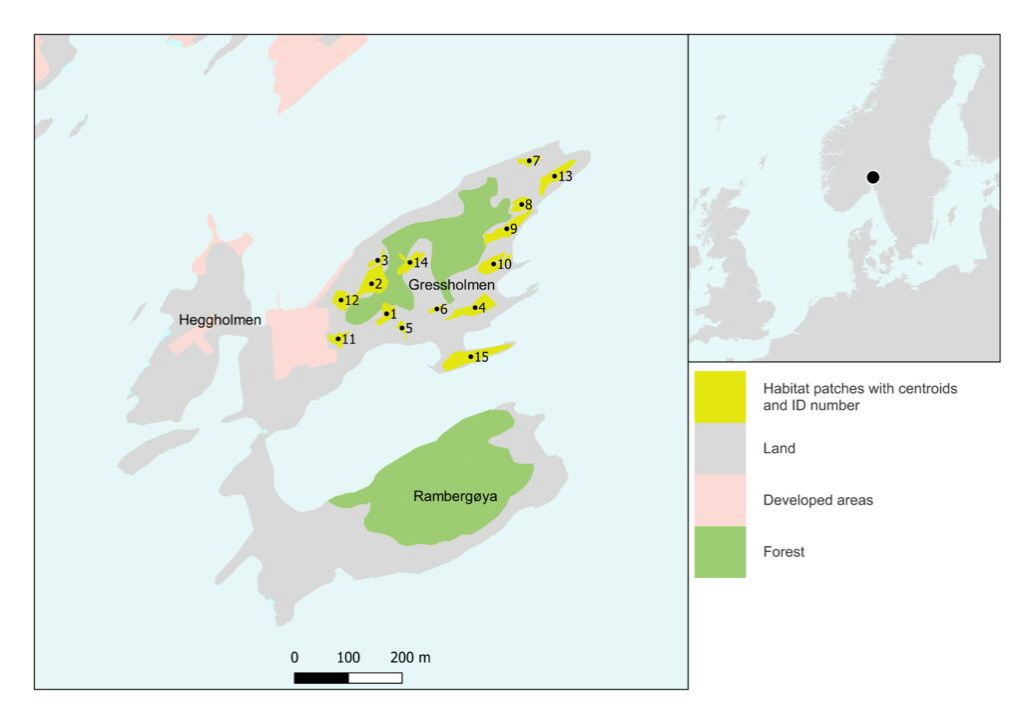
Study area on Gressholmen (left) and location in northern Europe (right). Numbered habitat patches are shown in yellow on Gressholmen Island.

The species were recorded in 15 patches of dry calcareous grassland on Gressholmen Island, situated in the inner Oslo fjord (Fig. 1). Habitat patches were identified through a combination of distribution modelling at 5 x 5 m resolution, interpretation of aerial photos, and in the field (Wollan et al. 2011). The mean patch size was 1078 m^2^, range 264 m^2^ to 2382 m^2^. Total habitat area, as the sum of all patch areas, was 48516 m^2^.

Humans have shaped and impacted the habitats on Gressholmen, probably for as long as the Oslo fjord has been settled. Today, the Island is artificially connected to two other Islands, Heggholmen and Rambergøya. Most of this Island trio is protected as nature reserves, but has previously housed a shooting range, an airport, a soap factory with connected houses, and a paint factory. It has likely been grazed by domestic animals while the Island was inhabited. From the 1970s until its eradication in 2007, a large rabbit population occupied the Islands and grazed the vegetation heavily. Several summerhouses, a small harbour, and a restaurant are still in use and the Islands are a popular destination for day-trips in the summer.

### Coordinates

59.882 and 59.887 Latitude; 10.717 and 10.727 Longitude.

## Taxonomic coverage

### Description

The dataset encompasses 49 habitat specialist vascular plant species. The list of habitat specialist species was developed, based on flora information of habitat requirements and distributional range, expert opinion, and field observations from a large number of polygons (see Evju and Sverdrup-Thygeson 2016). More vascular plant species were recorded in the first two years and are available upon request. Field surveyors used Norsk Flora (Lid and Lid 2005) and Gyldendals store nordiske flora (Mossberg and Stenberg 2012) for species identification. Species names were checked and updated using the Species Nomenclature Database (Norwegian Biodiversity Information Centre 2022) and matched with the best possible species name in GBIF. The list of taxa included follow the GBIF names as published in the dataset. The corresponding list of species names following the Norwegian Species Nomenclature Database is published on GitHub. Common names are given in Norwegian to provide an additional link between the different species lists, including to the original data collection file where some species names were outdated.

### Taxa included

**Table:** 

Rank	Scientific Name	Common Name
species	*Acinosarvensis* (Lam.) Dandy	Bakkemynte
species	*Androsaceseptentrionalis* L.	Smånøkkel
species	*Arabishirsuta* (L.) Scop.	Bergskrinneblom
species	*Ariaedulis* (Willd.) M.Roem.	Sølvasal
species	*Aspleniumruta-muraria* L.	Murburkne
species	*Avenulapratensis* (L.) Dumort.	Enghavre
species	*Carexcaryophyllea* Latourr.	Vårstarr
species	*Carlinavulgaris* L.	Stjernetistel
species	*Centaureascabiosa* L.	Fagerknoppurt
species	*Cerastiumsemidecandrum* L.	Vårarve
species	*Cotoneasterniger* (Fr.) Fr.	Svartmispel
species	*Cotoneasterscandinavicus* B.Hylmö	Dvergmispel
species	*Cynoglossumofficinale* L.	Hundetunge
species	*Drabaverna* L.	Vårrublom
species	*Dracocephalumruyschiana* L.	Dragehode
species	*Echiumvulgare* L.	Ormehode
species	*Epipactisatrorubens* (Hoffm.) Besser	Rødflangre
species	*Erysimumvirgatum* Roth	Berggull
species	*Filipendulavulgaris* Moench	Knollmjødurt
species	*Fragariaviridis* Duchesne	Nakkebær
species	*Geraniumsanguineum* L.	Blodstorkenebb
species	*Hypochaerismaculata* L.	Flekkgrisøre
species	*Inulasalicina* L.	Krattalant
species	*Lappulasquarrosa* (Retz.) Dumort.	Sprikepiggfrø
species	*Lepidiumcampestre* (L.) W.T.Aiton	Markkarse
species	*Ligustrumvulgare* L.	Liguster
species	*Linumcatharticum* L.	Vill-lin
species	*Lithospermumofficinale* L.	Legesteinfrø
species	*Myosotisramosissima* Rochel	Bakkeforglemmegei
species	*Myosotisstricta* Link ex Roem. & Schult.	Dvergforglemmegei
species	*Myosurusminimus* L.	Muserumpe
species	*Odontiteslitoralis* (Fr.) Fr.	Strandrødtopp
species	*Phleumphleoides* (L.) H.Karst.	Smaltimotei
variety	Poaalpinavar.alpina	Frøfjellrapp
species	*Poacompressa* L.	Flatrapp
species	*Polygonatumodoratum* (Mill.) Druce	Kantkonvall
species	*Potentillacrantzii* (Crantz) Beck ex Fritsch	Flekkmure
species	*Rhamnuscathartica* L.	Geitved
species	*Rosamajalis* Herrm.	Kanelrose
species	*Saxifragagranulata* L.	Nyresildre
species	*Saxifragaosloensis* Knaben	Oslosildre
species	*Saxifragatridactylites* L.	Trefingersildre
species	*Scleranthusperennis* L.	Flerårsknavel
species	*Seselilibanotis* (L.) W.D.J.Koch	Hjorterot
species	*Silenenutans* L.	Nikkesmelle
species	*Thymuspulegioides* L.	Bakketimian
species	*Veronicaarvensis* L.	Bakkeveronika
species	*Veronicaspicata* L.	Aksveronika
species	*Woodsiaalpina* (Bolton) Gray	Fjell-lodnebregne

## Temporal coverage

### Notes

07/07/2009 through 10/07/2009, 01/07/2019 through 16/08/2019, 04/06/2020 through 22/06/2020.

## Usage licence

### Usage licence

Open Data Commons Attribution License

### IP rights notes

This work is licensed under a Creative Commons Attribution (CC-BY) 4.0 License.

## Data resources

### Data package title

Presence-absence of plant habitat specialists in 15 patches

### Resource link

https://ipt.gbif.no/resource?r=geco-plant-habitat-specialists-15-patches

### Alternative identifiers

a99cf6c0-4eb2-476b-8414-a513f0925d86

### Number of data sets

2

### Data set 1.

#### Data set name

Presence-absence of plant habitat specialists in 15 patches: event

#### Data format

Darwin Core; tab separated text file

#### Description

We present three years of presence-absence data of 49 vascular plant species in 15 patches of dry calcareous grassland habitat (Lieungh et al. 2022). The first data file, event.txt, describes the surveys of each patch in each year as a sampling event. The file has 46 rows (15 patches times 3 years plus header row) and nine columns.

**Data set 1.** 

Column label	Column description
id	A unique identification number for each survey event per habitat patch, i.e. patch number one has three ids for each of the three years it was surveyed.
eventID	The same as id: unique identification number for each survey event per habitat patch.
samplingProtocol	A short description of the methods or protocols used during a sampling Event.
sampleSizeValue	A numeric value for a measurement of the size (time duration, length, area, or volume) of a sample in a sampling event.
sampleSizeUnit	The unit of measurement of the size (time duration, length, area, or volume) of a sample in a sampling event. Here: Area of the habitat patch in square metres
eventDate	The range of dates for the sampling event (survey of one patch in one year), given as year-month-startDay/endDay following the ISO 8601 date-time standard. Exact dates and times were not recorded, but we did record the start and end dates of fieldwork. One patch was surveyed within a day in the given date range.
startDayOfYear	The earliest integer day of the year on which the sampling event (survey) occurred (1 for 1 January, 365 for 31 December).
endDayOfYear	The latest integer day of the year on which the sampling event (survey) occurred (1 for 1 January, 365 for 31 December).
year	The year the habitat patch was surveyed.
locationID	Habitat patch number, given as patch-1, ..., patch-15. To connect back to data from the ARKO project, these patches correspond to polygons 35_1, ..., 35_15.
countryCode	Two-letter ISO 3166-1-alpha-2 country code. Here NO for Norway.
locationRemarks	Patch-year combination, where p1 corresponds to patch 1 and so on.
decimalLatitude	Latitude, in decimal degrees, of habitat patch centroid.
decimalLongitude	Longitude, in decimal degrees, of habitat patch centroid.
geodeticDatum	The spatial reference system (SRS) upon which the geographic coordinates given in decimalLatitude and decimalLongitude as based. EPSG:4326 is the EPSG codde for WGS84.
coordinateUncertaintyInMeters	The longest distance, in metres, from patch centroid coordinates to the patch polygon boundary. This measure overestimates uncertainty for most occurrences.
footprintWKT	A Well-Known Text (WKT) representation of the shape (footprint, geometry) of the patches, defining the Locations. These footprintWKTs are readable by geographic information systems (GIS) as vector polygon features.
footprintSRS	The geodetic datum or spatial reference system (SRS) of the footprintWKT. EPSG:4326 is the same as WGS84.

### Data set 2.

#### Data set name

Presence-absence of plant habitat specialists in 15 patches: occurrence

#### Data format

Darwin Core; tab separated text file

#### Description

The second data file, occurrence.txt, describes the species occurrences in three years of surveys, the last of which are re-surveys of a subset described in previous publications (Wollan et al. 2011, Bakkestuen et al. 2014, Evju et al. 2015, Evju and Sverdrup-Thygeson 2016, Evju et al. 2016).

The occurrence.txt file has 2206 rows, of which 999 are presence and 1206 absence observations, plus one header row.

**Data set 2.** 

Column label	Column description
id	A unique identification number for each observation, i.e. on observed presence or absence of a species in a patch in a year.
basisOfRecord	The specific nature of the data record, given by a standard label of one of the Darwin Core classes: here, human observation.
occurrenceID	A persistent, globally unique identifier for the Occurrence (as opposed to a particular digital record of the occurrence).
recordedBy	Name(s) of the observer who recorded the occurrence.
recordedByID	A unique identifier of the observer (in the recordedBy column), here their OrcID number.
organismQuantity	A number or enumeration value for the quantity of organisms. Here, either 0 for absent or "at least one" for present.
organismQuantityType	The type of quantification system used for the quantity of organisms. Here, number of indivuduals.
occurrenceStatus	A statement about the presence or absence of the taxon at the specified patch location and time.
occurrenceRemarks	A patch-year combination, where habitat patch 1 is shortened to p1 and so on, specifying the location and time of the occurrence.
eventID	A unique identifier for each survey event, to connect the occurrences to the sampling events in the event.txt file. There is one eventID per habitat patch per year, i.e. there are three eventIDs for patch 1 because it was surveyed three times.
year	The year the occurrence was recorded.
scientificName	The scientific name of the recorded taxon, in Latin and including author name, mapped to the GBIF taxonomic backbone from the closest match in the Norwegian Species Nomenclature Database.
kingdom	The full scientific name of the kingdom in which the taxon is classified, here Plantae.
taxonRank	The taxonomic rank of the most specific name in the scientificName, for exampe, species or variety. Most of the occurrences were recorded on species level.
vernacularName	Common name in Norwegian. Provides a link back to the original data collection sheet and to the Norwegian Species Nomenclature Database.

## Additional information

Additional data can be found on GitHub: https://github.com/evalieungh/gressholmen_data
